# Differential modulation of nociceptive versus non-nociceptive synapses by endocannabinoids

**DOI:** 10.1186/1744-8069-9-26

**Published:** 2013-06-01

**Authors:** Alexandra Higgins, Sharleen Yuan, Yanqing Wang, Brian D Burrell

**Affiliations:** 1 Division of Basic Biomedical Sciences, Sanford School of Medicine, University of South Dakota, Vermillion, SD, 57069, USA

**Keywords:** Endocannabinoid, Nociception, GABA, Leech, Invertebrate, Synapse

## Abstract

**Background:**

Although a number of clinical and preclinical studies have demonstrated analgesic effects of cannabinoid treatments, there are also instances when cannabinoids have had no effect or even exacerbated pain. The observed pro-nociceptive effects appear to be due to cannabinoid-induced disinhibition of afferent synaptic input to nociceptive circuits. To better understand how cannabinoid-mediated plasticity can have both pro- and anti-nociceptive effects, we examined the possibility that cannabinoids differentially modulate nociceptive vs. non-nociceptive synapses onto a shared postsynaptic target. These experiments were carried out in the central nervous system (CNS) of the medicinal leech, in which it is possible to intracellularly record from presynaptic nociceptive (N-cell) or pressure-sensitive (P-cell) neurons and their shared postsynaptic targets.

**Results:**

The endocannabinoid 2-arachidonoyl glycerol (2AG) elicited significant long-lasting depression in nociceptive (N-cell) synapses. However, non-nociceptive (P-cell) synapses were potentiated following 2AG treatment. 2AG-induced potentiation of non-nociceptive synapses was blocked by the TRPV antagonist SB366791, suggesting involvement of the same TRPV-like receptor that has already been shown to mediate endocannabinoid-dependent depression in nociceptive inputs. Treatment with the GABA receptor antagonist bicuculline also blocked 2AG-induced potentiation, consistent with the idea that increased synaptic signaling was the result of endocannabinoid-mediated disinhibition. Interestingly, while bicuculline by itself increased non-nociceptive synaptic transmission, nociceptive synapses were depressed by this GABA receptor antagonist indicating that nociceptive synapses were actually excited by GABAergic input. Consistent with these observations, GABA application depolarized the nociceptive afferent and hyperpolarized the non-nociceptive afferent.

**Conclusions:**

These findings show that endocannabinoids can differentially modulate nociceptive vs. non-nociceptive synapses and that GABAergic regulation of these synapses plays an important role in determining whether endocannabinoids have a potentiating or depressing effect.

## Background

Endogenous cannabinoids (endocannabinoids or eCBs), such as anandamide and 2-arachidonoyl glycerol (2AG), are lipid neurotransmitters found throughout the central nervous system of both vertebrates and invertebrates that modulate a number of behavioral processes including appetite, cognition, emotion, sensory processing and nociception [1,2]. In many regions of the CNS, the primary physiological effect of eCBs is depression of synaptic transmission that can be short-term (milliseconds to seconds) or long-term (tens of minutes to hours) [3]. eCBs can depress both excitatory and inhibitory synapses, so that from a functional standpoint these neurotransmitters can be bi-directional modulators of neural circuits depending on whether there is depression of an excitatory pathway (decreasing circuit activity) or depression of an inhibitory pathway (increasing circuit activity via disinhibition). This is a significant consideration in terms of development of eCB-based pharmacotherapies because treatments focused on depression of excitatory synapses may produce undesired effects through depression of inhibitory synapses. The importance of this issue has become apparent in the potential use of cannabinoid-based treatments for chronic pain. In a recent study, cannabinoids were found to reduce GABAergic and glycinergic inhibitory signaling in the spinal cord that contributed to secondary hyperalgesia due to disinhibition of afferent synaptic input to spinal pain circuits [4]. However, it was not known whether this pro-algesic effect occurred at the nociceptive afferent synapses (Aδ and C fibers) or at non-nociceptive afferent synapses (Aβ fibers). Non-nociceptive afferents can have input to pain circuits in the spinal cord as a result of central sensitization and can potentially contribute to chronic pain conditions [5].

**Figure 1:**
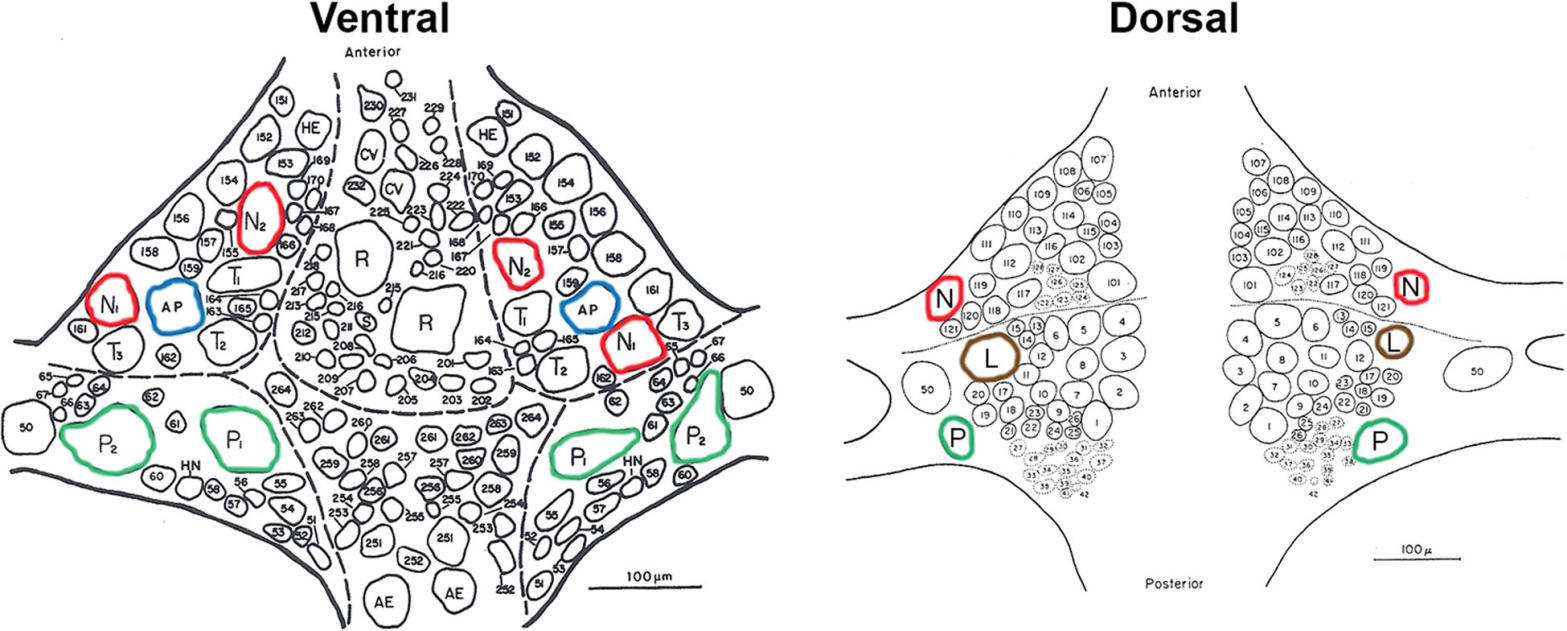
**Ventral and dorsal aspects of a single ganglion from the leech CNS (modified from [**[13]**]).** Neurons used in this study have been outlined in red (N-cell), green (P-cell), blue (AP-cell) and brown (L motor neuron).

To address this issue, the CNS of the medicinal leech (*Hirudo verbana*) was used to test the effects of eCBs on nociceptive vs. non-nociceptive synapses. The leech is a useful model system in which to carry out these studies because it has a well-defined nervous system in which the identity of nociceptive and non-nociceptive neurons are known as well as their synaptic targets. The leech CNS consists of a chain of segmentally-arranged ganglia, each with its own set of sensory, motor and local and intersegmental interneurons. Similar to mechanosensation in mammals, the leech receives input from three distinct catergories of sensory neurons innervating the skin: rapidly-adapting touch cells (T-cells), slow-adapting pressure-sensitive neurons (P-cells) and two types of nociceptive neurons (N-cells), one that is a mechanical and the other a polymodal nociceptor that responds to mechanical, thermal and chemical stimuli [6-8]. The cell bodies for each type of somatosensory neuron are located in the segmental ganglia and can be easily identified based on their size, position within each ganglion (see Figure 1) and electrophysiological properties. Furthermore, a number of the postsynaptic targets of these afferents are known and in many cases are shared by all three types of sensory neurons [9]. Therefore, it is possible to selectively carry out dual intracellular recordings of both the pre- and postsynaptic neurons in these nociceptive vs. non-nociceptive synapses. This capacity to carry out detailed studies from identifiable neurons and their synapses has already been used to develop a detailed understanding the cellular signal mechanisms mediating eCB-dependent depression of nociceptive synapses in the leech [10-12].

The leech CNS is known to contain the eCBs 2AG and anandamide with 2AG being the more abundant transmitter [14]. Furthermore, 2AG has been shown to mediate long-term depression (LTD) in leech nociceptive synapses that are remarkably similar to mammalian eCB-LTD in terms of cellular mechanisms [3,10]. Like other protostomal invertebrates, the leech lacks orthologues to the vertebrate cannabinoid receptors CB1 and CB2 [2]. However, transient potential receptor vanilloid (TRPV) channels can also act as cannabinoid receptors [15-18] and previous pharmacological studies from our laboratory indicate that a leech TRPV-like receptor mediates 2AG-induced depression in nociceptive synapses [10].

In the present study, the effects of 2AG on nociceptive synapses (those made by the N-cells) versus non-nociceptive synapses (those made by the P-cells) were compared in which both afferent types converged on the same postsynaptic target (the longitudinal or “L” motor neuron). 2AG elicited depression at nociceptive synapses, but potentiation at non-nociceptive synapses. This 2AG-mediated potentiation of the non-nociceptive synapses required functional GABA receptors, indicating eCB-induced increases in synaptic signaling were the result of synaptic disinhibition.

## Results

### Effects of 2AG on nociceptive vs. non-nociceptive synapses

**Figure 2:**
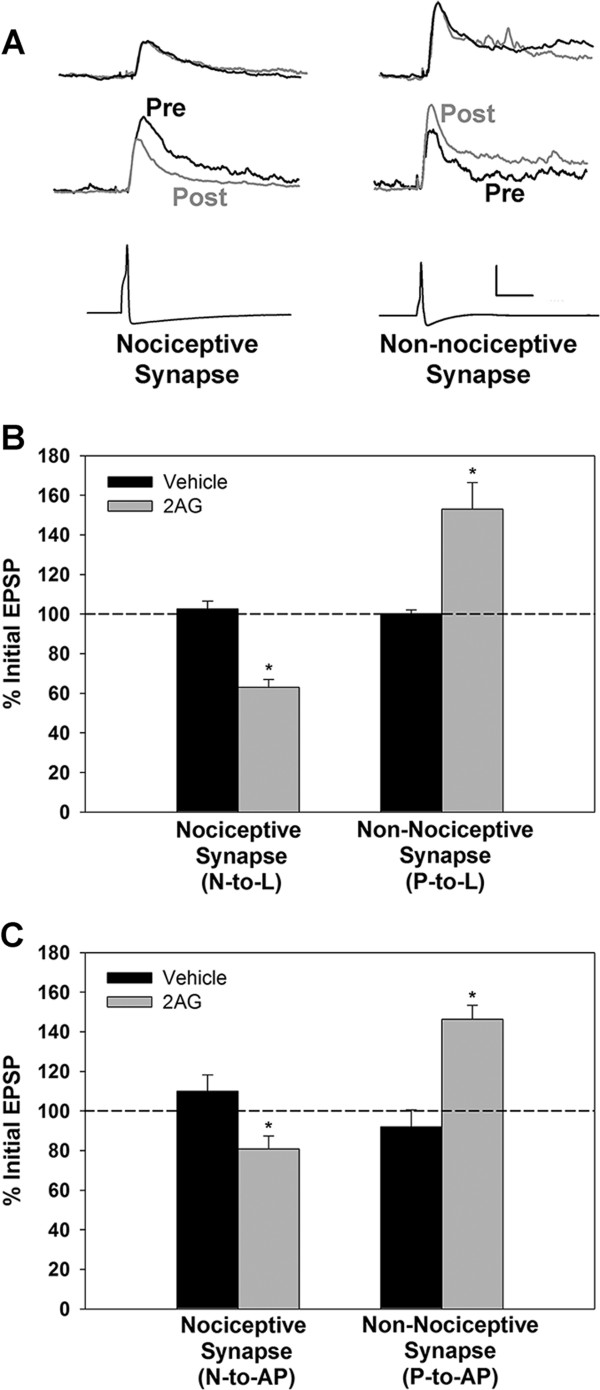
**Differential endocannabinoid modulation of nociceptive vs. non-nociceptive synapses.** (**A**) Sample traces from experiments using nociceptive (N-to-L, left) and non-nociceptive (P-to-L, right) synapses. Top EPSP traces are from control experiments in which pre- and post-test recordings (black and grey traces, respectively) were made 75 mins apart without 2AG treatment. Middle EPSP traces are from experiments in which pre- and post-test recordings were made in 2AG-treated ganglia. Bottom traces are action potentials from the presynaptic N-cell (left) or P-cell (right). Vertical calibration bar is 2mV EPSP traces and 50 mV for action potential traces. Horizontal calibration bar is 50 msec for all. (**B**) Bar graph showing that 2AG depressed the N-to-L synapse, but potentiated the P-to-L synapse. (**C**) Bar graph showing that 2AG depressed the N-to-AP synapse, but potentiated the P-to-AP synapse. Asterisks indicate statistically significant difference relative to the vehicle control group (see Results section for details).

For most of the experiments in this study, excitatory post-synaptic potentials (EPSPs) were recorded from synapses made by either the lateral (polymodal) nociceptive (N-) cell or one of the pressure-sensitive (P-) cells onto the L motor neuron (Figure 1). The L motor neuron innervates the longitudinal muscles and is active during the defensive withdrawal reflex, whole-body shortening [19]. This motor neuron receives monosynaptic, glutamatergic input from the both the P- and N-cells [10,20]. Previous studies have shown that 2AG (60 μM for 15 mins) induced LTD in nociceptive synapses made by the lateral N-cell onto the L motor neuron (Figure 2A, B; data re-presented from [10]; t=6.45, p≤0.0001, n=5). In contrast, the non-nociceptive P-to-L synapse was actually potentiated 1 hr following bath application of 60–100 μM 2AG for 15 min (N=10) relative to control synapses in which 2AG application was omitted (Figure 2A, B; t=3.37, p≤0.005, n=10). Potentiation did not appear to be due to postsynaptic changes in intrinsic excitability given that input resistance (IR) in the L motor neuron was unchanged (post-test IR was 100.6±2.9% of pre-test levels in the 2AG-treated group and 103.2±3.7% in the vehicle control group). The effects of 60 and 100 μM 2AG on the P-to-L synapse were indistinguishable with 60 μM 2AG producing a 48.2±10.4% increase (n=4) and 100 μM producing a 56.1±22.1% (n=6) increase in EPSP amplitude (independent *t*-test p=0.79); therefore the data from these two concentrations were combined. N-to-L synapses treated with 100 μM 2AG still underwent depression (64.6±10.3% of initial EPSP amplitude; n=3) that was no different from the effects observed at 60 μM (63±4%; independent *t*-test p=0.87). These results indicate that the potentiating effect of 100 μM 2AG on the non-nociceptive synapse was not a consequence of the concentration of 2AG used.

To determine if these opposing effects of 2AG were a feature of other nociceptive and non-nociceptive synapses, N- and P-cell input to the anterior pagoda (AP) cell was also examined. Although the function of the AP cell is not known, the monosynaptic, glutamatergic P-to-AP synapses have been used to study a variety of forms of synaptic plasticity in the leech [21-25]. However, the N-to-AP synapse had not been previously characterized and this was done prior to conducting the 2AG experiments. In general the N-to-AP synapse was quite similar to the N-to-L connection. N-to-AP was substantially reduced by CNQX (20 μM) indicating glutamatergic transmission (Additional file 1: Figure S1A). In experiments with high MgCl_2_ saline (15 mM), which blocked all chemical synaptic transmission [20], the majority of the N-to-AP EPSP was eliminated except for a small (<1 mV) early component that appeared to be a rectifying electrical synapse between the N-and AP-cell (Additional file 1: Figure S1B). This putative electrical EPSP was also observed in the CNQX experiments. Experiments with high divalent saline (18 mM MgCl_2_/15 mM CaCl_2_) eliminated later components of the N-to-AP EPSP indicating the presence of polysynaptic elements (Additional file 1: Figure S1C). While positive current associated with the N-cell action potential did propagate to the AP via electrical coupling, negative current injected into the N-cell did not spread to the AP (data not shown). Interestingly, evidence of electrical coupling was also observed in the AP-to-N direction. Injection of 500 msec current pulses showed that negative current, but not positive current, could spread from the AP to the N-cell (Additional file 1: Figure S1D).

In terms of eCB modulation, 2AG (100 μM) depressed N-to-AP synapses (n=9 and 5 respectively; t=2.91, p≤0.05), but potentiated P-to-AP synapses relative to controls (n=6 and 5 respectively; t=2.36, p≤0.05) identical to N- and P-cell inputs to the L motor neuron (Figure 2C). No obvious changes in postsynaptic IR were observed during the P-to-AP (2AG group=111.5±3.2%, control=106.8±5.6) or N-to-AP (2AG group=104.6±3.8%, control=97.7±6.2%) synapses.

**Figure 3:**
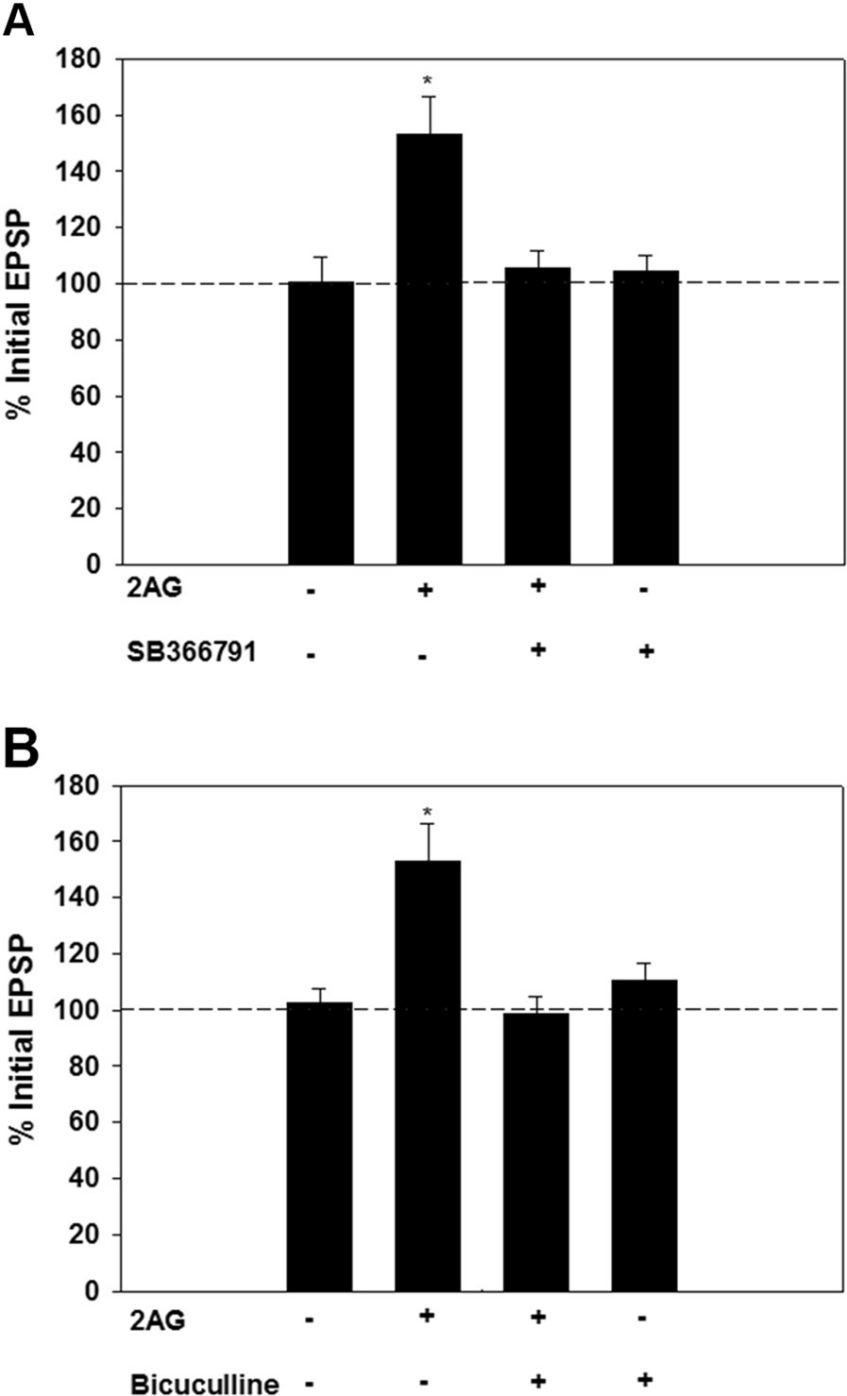
**Role of the leech TRPV-like receptor and GABA during 2AG-mediated synaptic potentiation.** (**A**) The TRPV1 antagonist, SB366791 (10 μM), prevents potentiation of the non-nociceptive P-to-L synapses that is normally observed following 2AG treatment. Asterisks indicate statistically significant difference relative to the vehicle control group (see Results section for details). (**B**) Role of GABA signaling during 2AG-mediated synaptic potentiation. Pretreatment of synapses with the GABA receptor antagonist bicuculline (100 μM), prevented 2AG-induced potentiation of the non-nociceptive N-to-L synapse. Asterisks indicate statistically significant difference relative to the vehicle control group (see Results section for details).

To test whether 2AG-induced potentiation of the non-nociceptive P-cell synapses is mediated by a TRPV-like receptor, experiments were carried out using the selective TRPV1 antagonist SB366791 (10 μM), which blocks eCB-mediated LTD in leech nociceptive synapses [10]. When SB366791 was co-applied with 2AG, potentiation of the non-nociceptive P-to-L synapse was prevented (Figure 3A; n=6). Application of SB366791 alone had no effect on the P-to-L EPSP (N=5). One-way ANOVA of the 2AG+SB366791, SB366791, 2AG and vehicle control groups showed a significant treatment effect (F_2,18_=6.01, p≤0.05). Post-hoc analysis showed that the 2AG group was statistically different from the control group (p≤0.05), but that the 2AG+SB366791 and SB366791 groups were not. Postsynaptic IR was unchanged in the 2AG+SB366791 (110.1±4.6% of pre-test levels), SB366791-only (101.7±5.6%) and vehicle control groups (101±3.9%; n=6). These findings indicate that the difference in the effects of 2AG on nociceptive vs. non-nociceptive synapses is not due to activation of different endocannabinoid receptor.

### Role of GABA receptors during 2AG-induced potentiation

eCBs are known to depress inhibitory synaptic transmission and eCB-induced disinhibition has been shown to increase synaptic signaling within spinal nociceptive circuits, although the identity of the presynaptic input(s) being enhanced was not known [4]. Therefore, we examined the potential role of GABA-A receptors during 2AG-induced potentiation of the non-nociceptive synapse in the leech. The leech CNS contains GABAergic neurons and the GABA-A receptor antagonist bicuculline has been shown to disinhibit other circuits that utilize P-cell input [26-28]. To test whether functional GABA-A receptors are required for 2AG-mediated potentiation, ganglia were pre-treated with bicuculline (100 μM) followed by the pre-test measurements of the P-to-L EPSP, 15 min application of 2AG, washout for 60 mins and finally post-test EPSP measurements. The 2AG treatment that normally produces potentiation was blocked in the non-nociceptive P-to-L synapses that had undergone bicuculline+2AG treatment (Figure 3B; n=5). P-to-L synapses that were pre-treated with bicuculline, but subsequent 2AG treatment omitted, were unchanged between the pre- and post-tests (Figure 3B, n=5). One-way ANOVA of the 2AG+bicuculline, bicuculline, 2AG and vehicle control group showed a significant effect of treatment (F_2,24_=7.13, p≤0.005). Post-hoc analysis showed that the 2AG group was statistically different from the control group (p<0.01) and that the 2AG+bicuculline and bicuculline groups were not. No change in postsynaptic input resistance was observed in the 2AG+bicuculline (104±4.4%) or bicuculline only group (96±4.6%). These findings support the hypothesis that 2AG-induced potentiation of non-nociceptive synapses is mediated by disinhibition as a result of a decrease in GABAergic input.

**Figure 4:**
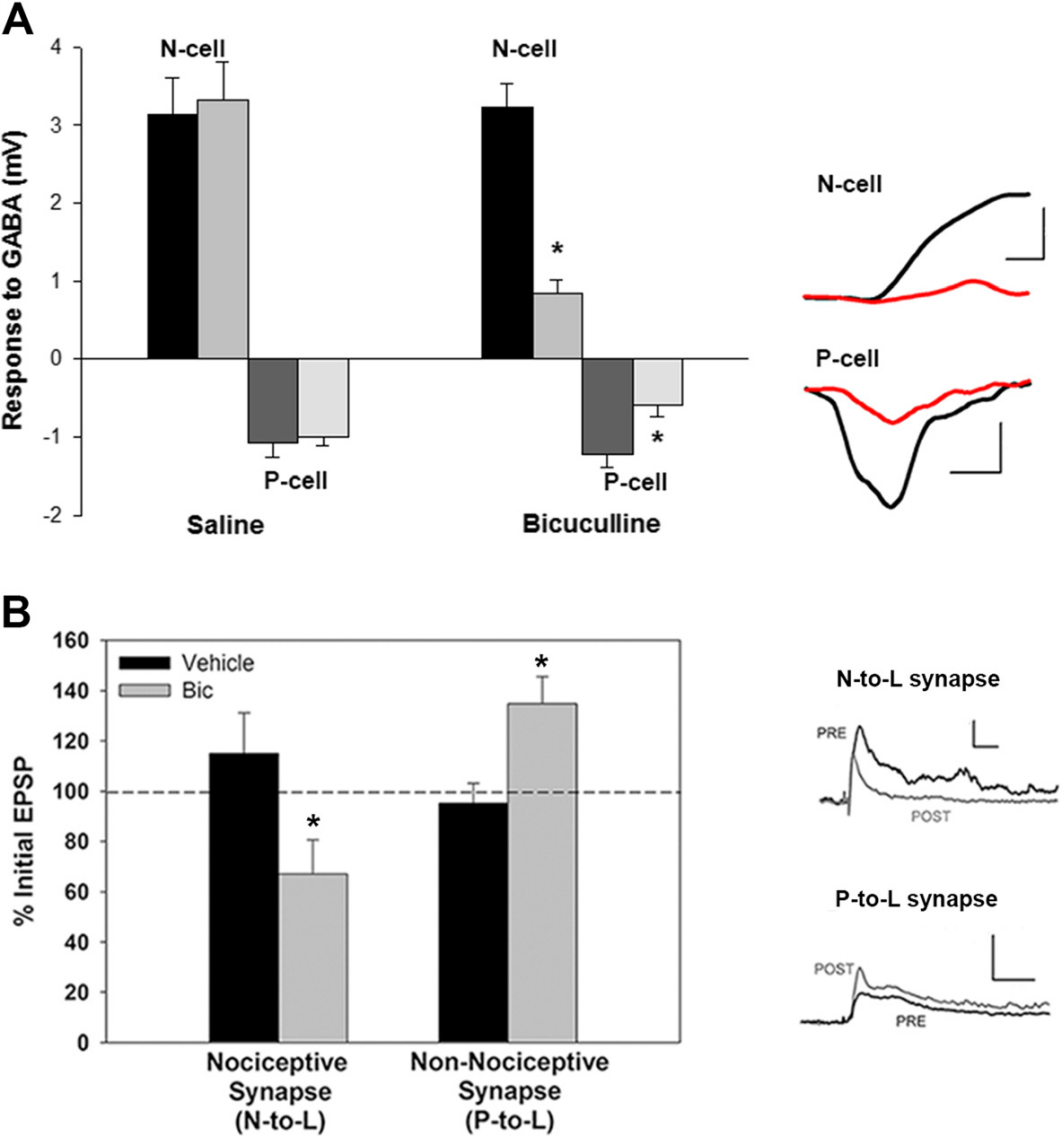
**Opposing effects of GABA on nociceptive vs. non-nociceptive afferents and their synapses.** (**A**) Response of nociceptive (N-cell) and non-nociceptive (P-cell) afferents to GABA. GABA elicited depolarization in the N-cells and hyperpolarization in the P-cells (scale bars are, respectively, 2 mV/500 msec and 0.5 mV/1000 msec). Both responses were inhibited by subsequent treatment 15 mins with bicuculline (100 μM) and this decrease was not observed when a 15 mins saline treatment was used in place of bicuculline. (**B**) Acute bicuculline treatment depresses the nociceptive (N-to-L) synapse, but enhances the non-nociceptive (P-to-L) synapse. Scale bars are, respectively, 2 mV/50 msec and 5 mV/50 msec. Asterisks indicate statistically significant difference relative to the vehicle control group (see Results section for details).

If 2AG potentiates non-nociceptive synapses due to a decrease in GABAergic input, why are nociceptive synapses not similarly disinhibited? GABA has been observed to depolarize the lateral N-cells used in these experiments [29] so it is possible that GABA excites rather than inhibits the nociceptive synapse. To confirm this earlier finding and to determine what effect GABA has on the P-cell, intracellular recordings of the N- and P-cells were made in response to GABA delivered via puffer electrodes. GABA reliably elicited depolarization in the lateral N-cells, consistent with earlier findings, but produced hyperpolarization in the P-cells (Figure 4A). Both the N-cell (n=6) and P-cell (n=3) responses were significantly inhibited following 15 min bath-application of 100 μM bicuculline (Figure 4A; N-cell, paired *t*-test t=8.80, p≤0.001; P-cell, t=4.56, p≤0.05). No changes in N-cell (n=5) or P-cell (n=5) GABA response were observed in control experiments in which responses were recorded prior to and then 15 mins following treatment with normal saline (Figure 4A; N-cell, paired *t*-test t=1.65, p>0.05; P-cell, t=0.81, p>0.05).

Next, P-to-L and N-to-L EPSPs were recorded prior to and then during acute bicuculline treatment (100 μM for 10–15 mins). As expected, the P-to-L EPSP amplitude increased during bicuculline treatment (n=7) compared to control synapses (n=5); that is, disinhibition of the non-nociceptive synapse was observed (Figure 4B; t=2.71, p≤0.05). However, bicuculline treatment actually decreased the amplitude of the N-to-L EPSP (n=8) compared to control synapses (n=5) suggesting that GABA has a tonic excitatory effect on this nociceptive synapse (Figure 4B; t=2.35, p≤0.05). No change in postsynaptic input resistance was observed during either the P-to-L synapse (bicuculline=96.7±4.9, control=105.7±6.4%) or N-to-L synapse (bicuculline=107.7±2.2%, control=98.7±3%) experiments. These findings are consistent with the idea that this nociceptive synapse does not undergo 2AG-induced disinhibition, at least in part, because the presynaptic neuron is excited, not inhibited, by GABA.

## Discussion

The endocannabinoid 2AG was found to differentially modulate nociceptive vs. non-nociceptive synapses in the leech, depressing the former and potentiating the latter. 2AG-induced potentiation of non-nociceptive synapses was blocked by the TRPV1 inhibitor SB366791, suggesting that the involvement of a TRPV-like receptor. In mammals, the TRPV1 receptor has been shown to be an important cannabinoid receptor that mediates synaptic depression in a variety of brain regions [16-18,30]. Protostomal invertebrates lack orthologues to the vertebrate CB1/CB2 receptors [2] and we have previously proposed that TRP channels, such as TRPV, may represent the earliest endocannabinoid receptors in the animal kingdom [10,11]. One critical difference between the nociceptive and non-nociceptive synapses is that while N-cells appear to possess TRPV-like receptors, P-cells do not [8]. This would indicate that the effects of 2AG on P-cell synapses are indirect and mediated by an unknown, 2AG-sensitive neuron. It is unlikely that the postsynaptic L motor neuron is being directly modulated by 2AG because it too lacks TRPV-like receptors, although it does appear to be capable of synthesizing 2AG [10].

**Figure 5:**
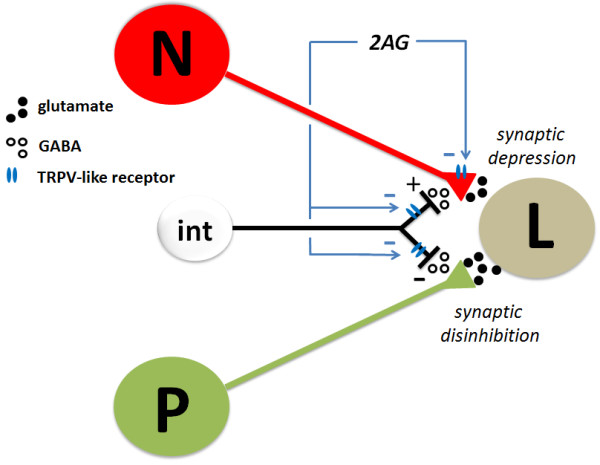
**Hypothetical model for opposing effects of 2AG on nociceptive (N) versus non-nociceptive (P) synapses.** Both N- and P-cells have input onto the same postsynaptic targets (in this example, the L motor neuron) via glutamatergic synapses. Based on previous studies [10], 2AG directly depresses the nociceptive synapse via a TRPV-like receptor that reduces presynaptic neurotransmitter release. In the present study, 2AG was observed to potentiate the non-nociceptive synapse via an indirect mechanism in which the eCB reduces inhibitory input from an unknown GABAergic interneuron (int). This 2AG-mediated disinhibition appears to be mediated by the TRPV-like receptor as well. Nociceptive synapses are “protected” from this disinhibitory because they are depolarized by GABA. The same GABAergic interneuron is shown to act on both the N- and P-cells for diagrammatic purposes and it is not known whether these afferents receive GABAergic input from a common source or from distinct interneurons.

Potentiation of non-nociceptive synapses was blocked when the GABA-A receptor antagonist bicuculline was applied. Bicuculline by itself was found to enhance non-nociceptive synaptic transmission indicating that this synapse is tonically regulated by inhibitory GABAergic input, consistent with previous studies of P-cell signaling [26,27]. From these results, we propose a model in which 2AG depresses inhibitory synaptic transmission from an unknown GABAergic neuron(s) onto P-cell synapses resulting in disinhibition of these non-nociceptive synapses (see Figure 5). When GABAergic input was reduced by bicuculline pre-treatment, the ability of 2AG to elicit potentiation via this proposed disinhibition was occluded. It should be noted that the specificity of both SB366791 and bicuculline as antagonists has not been directly tested on isolated leech versions of the TRPV and GABA receptors. Therefore, the possibility exists that the effects of both of these pharmacological agents are due to actions not associated with either of these receptors.

The fact that 2AG did not potentiate nociceptive synapses appears to be due to differences in how GABA affects nociceptive vs. non-nociceptive synapses (Figure 5). The same bicuculline treatment that disinhibited non-nociceptive synapses actually decreased N-cell synaptic transmission suggesting that GABA has an excitatory effect on these nociceptive synapses. This is supported by the observation that GABA applied to the soma depolarizes the lateral N-cell [29]. In the present study, the ability of GABA to depolarize the N-cell was replicated and extended by showing that this GABA-induced depolarization could be inhibited by bicuculline. Furthermore, direct application of GABA to the P-cell was found to elicit hyperpolarization that was also inhibited by bicuculline. The excitatory effects of GABA on the N-cell are likely the result of elevated intracellular Cl^-^ concentration so that the Cl^-^ equilibrium potential is depolarized relative to the resting potential. Thus, activation of ionotropic GABA receptors would cause a Cl^-^ efflux, depolarizing the N-cell. It well-established that GABA can depolarize nociceptive afferents in the spinal cord as a result of elevated intracellular Cl^-^ concentrations/Cl^-^ equilibrium potential (E_Cl_) [31]. GABA-mediated depolarization has been shown to increase synaptic transmission in both the brain and the spinal cord circuits [32-35], although there are also instances when GABA-mediated depolarization inhibits synaptic transmission via current shunts or Na^+^ channel inactivation [31,35].

It is not clear whether GABAergic modulation of the P-to-L synapse is being exerted on the presynaptic or postsynaptic neuron (or both). Leech motor neurons do receive inhibitory GABAergic input [26,36], but no changes in postsynaptic input resistance were observed during any of the 2AG experiments, which would argue against a postsynaptic mechanism. Furthermore, the fact that N- and P-cell synapses onto the same postsynaptic target (L motor neuron or AP cell), but underwent opposite changes following 2AG treatment would also support a presynaptic mechanism for both eCB-induced potentiation and depression. Previous studies have been supportive of a presynaptic mechanism mediating 2AG-induced depression of nociceptive (N-to-L) synapse [10,11]. Nevertheless, the possibility of localized, synapse-specific changes in the postsynaptic neuron cannot be eliminated and additional studies are required to further assess the role of pre- vs. postsynaptic modulation during eCB-mediated synaptic plasticity.

The results from this study address important issues regarding the modulatory effects of eCB at the microcircuit level. It is well-established that eCBs depress inhibitory (as well as excitatory) synaptic transmission and that such eCB-mediated disinhibition is likely to have important consequences in terms of neural circuit function [16,37-42]. For example, eCB-induced disinhibition has been observed to lower the threshold for initiating LTP [43-45]. However, this is the first time, to our knowledge, that eCB-induced increases in evoked synaptic transmission via disinhibition have been directly observed. Furthermore, by using the well-described CNS of the leech it was possible to observe opposing synaptic effect of eCBs (depression vs. potentiation) on distinct afferent inputs that converge onto a shared postsynaptic target. This allowed us to address the issue as to why eCB-induced disinhibition does not lead to increased synaptic signaling in more neurons given the widespread nature of GABAergic regulation of synaptic transmission. We found evidence that while some synapses were sensitive to eCB-mediated disinhibition; others were “protected” from such modulation because they were not inhibited by GABA. In fact, GABA appeared to have an excitatory effect on these synapses based on results from experiments in this study and those from Sargeant et al. [29], presumably as a result of elevated intracellular Cl^-^ levels. This would suggest that the Cl^-^ gradient in neurons plays a critical in regulating the effects of eCBs on synaptic signaling. Such a mechanism has already been suggested by Christie and Mallet [46].

These findings may contribute to understanding why there is such conflicting evidence from both animal model and clinical studies regarding the efficacy of cannabinoid based analgesic therapies [46-50]. In some clinical studies, cannabinoids were found to be ineffective and could even enhanced pain associated with evoked mechanical stimuli resulting in mechanical hyperalgesia or allodynia [4,46,51]. One explanation for these pro-nociceptive effects is that eCBs have been shown to depress inhibitory synapses in the spinal cord, thereby disinhibiting spinal pain circuits, which contributes to the development of secondary mechanical hyperalgesia [4]. It is possible that eCBs selectively enhance synaptic input from afferents that contribute to mechanical hyperalgesia or allodynia, such as Aβ fibers [5], but depress synaptic input from nociceptive afferents, such as Aδ or C fibers [50]. As stated above, these synaptic potentiation vs. depression effects of eCBs may be due to whether GABA inhibits or excites the relevant afferent [46]. Although the present study is focused on changes in afferent signaling, inhibitory and excitatory interneurons in the dorsal horn can also undergo shifts in the E_Cl_ that lead to GABA-elicited depolarization during neuropathic pain [52,53]. Changes in GABAergic signaling onto these neurons may also impact how cannabinoid-based treatments affect these nociceptive neural circuits.

## Conclusions

Nociception is a fundamental sensory process that exhibits considerable evolutionary conservation between vertebrates and invertebrates [54,55]. The leech, in particular, provides a useful model system in which to study the basic physiological processes related to nociception. It possesses afferents innervating the skin that demonstrate clear nociceptive properties and share many features with vertebrate polymodal nociceptors including a high threshold for both mechanical and thermal (>40°C) stimuli as well as sensitivity to noxious chemical stimuli [8]. These nociceptors are clearly distinguishable from the lower threshold non-nociceptive mechanosensory neurons and it is possible to carry out detailed recordings from nociceptive vs. non-nociceptive synapses.

The findings from the present study suggest that the effectiveness of cannabinoid-based analgesic therapies is likely to depend on the type of nociception that is being experienced. As already stated, there is evidence from both preclinical and clinical studies that eCBs can have both pro- and anti-nociceptive effects [4,46]. Interestingly, there is also evidence to suggest that central TRPV receptor activation can have opposing effects on spinal nociceptive circuits in rodents mediating both synaptic disinhibition that resulted in allodynia [56] and persistent depression of C fiber evoked EPSPs [57-59]. Cannabinoid-based therapies may be appropriate for conditions that result from spontaneous activity from nociceptive afferents, such neuropathy-associated chronic pain [49,60]. On the other hand, conditions that are dominated by mechanical hyperalgesia and/or allodynia may be insensitive to or even exacerbated by cannabinoid-based treatments due to the potential involvement of non-nociceptive afferents [5]. Additional studies using the leech CNS, at both the synaptic and behavioral level, may contribute to better understanding the pro- vs. anti-nociceptive effects of eCBs.

## Methods

### Animal preparation

Leeches (*Hirudo verbana*) were obtained from commercial suppliers (Leeches USA, Westbury, NY and Niagara Leeches, Cheyenne, WY) and maintained in artificial pond water (0.50g/L H_2_O *Hirudo* salt) on a 12 hour light/dark cycle at 18°C. Ganglia were dissected and pinned in a recording chamber with constant perfusion of normal leech saline (≈1.5 ml/min). All dissections and recordings were carried out in normal leech saline (110 mM NaCl, 4 mM KCl, 1.8 mM CaCl_2_, 1 mM MgCl_2_, 5 mM NaOH, and 10 mM HEPES, pH=7.4). Drugs were dissolved in leech saline from stock solutions and final concentrations were made just prior to respective experiments. The following drug was obtained from Tocris (Ellisville, MO): 2-arachidonoyl glycerol (2AG). Drugs obtained from Sigma-Aldrich (St. Louis, MO) included CNQX, dimethyl sulfoxide (DMSO), and bicuculline.

### Electrophysiology

Techniques used in this study have been described in detail in [10]. Briefly, current clamp (bridge balanced) intracellular recordings were carried out using sharp glass microelectrodes (tip resistance 35–40 MΩ) made from borosilicate capillary tubing (1.0 mm OD, 0.75 mm ID; FHC, Bowdoinham, ME) using a horizontal puller (Sutter Instruments P-97; Novato, CA). Microelectrodes were filled with 3M potassium acetate. Manual micropositioners (Model 1480; Siskiyou Inc., Grants Pass, OR) were used to impale individual neurons during experiments. Current was delivered to electrodes using a multi-channel programmable stimulator (STG 1004; Multi-Channel Systems; Reutlingen, Germany) and the signal was recorded using a bridge amplifier (BA-1S; NPI, Tamm, Germany) and digitally converted for analysis (Axoscope; Molecular Devices, Sunnyvale, CA).

The presynaptic lateral nociceptive (N) and pressure (P) cells and the postsynaptic longitudinal (L) motor neuron and anterior pagoda (AP) cell were identified based on their position with the ganglion (Figure 1), size, and characteristic electrophysiological properties (size and shape of action potential). L motor neuron identification could be confirmed by recording from the electrically coupled contralateral L motor neurons and observing synchronous activity [61]. For experiments utilizing N-to-L and P-to-L synapse recordings, the ganglion was pinned dorsal side up so that the L motor neurons could be located on the dorsal side along with access to the lateral-most N- and P-cells. For N-to-AP and P-to-AP synapse recordings, the ganglion was pinned ventral side up. Following pre-test recordings of the excitatory postsynaptic potentials (EPSPs), the ganglion was superfused with 2AG for 15 minutes and then returned to normal saline. In vehicle control experiments, 2AG was replaced with saline containing 0.01% DMSO. After one hour, the EPSP was retested (post-test). Separate electrode impalements of the same presynaptic and postsynaptic neuron were made for pre- and post-test recordings. Chronic intracellular recordings of these neurons were not carried out because this results in progressive rundown of the EPSP within 10–15 mins most likely due to damage caused by movements of the tissue during the electrode impalement (there are muscle fibers and connective tissue present in the leech CNS). Input resistance was recorded at the pre- and post-test level and only consistent, stable recordings were included in the data analysis (see Results section). The peak EPSP amplitude was recorded every 10 seconds and calculated by averaging 5–10 EPSP (pre- or post-test) sweeps.

For N-to-AP characterization experiments, the ganglion was pinned ventral side up as the lateral N-cell and AP-cell were both located on the ventral side. Treatments for the set of characterization experiments (high Mg^2+^ saline, high divalent saline, and CNQX) were perfused for 10 to 15 minutes between pre-test and post-test recordings while controls were carried out in constant perfusion of normal leech saline. Coupling experiments were performed by injecting single 500 msec hyperpolarizing current pulses with increasing amounts of current into the AP- or N-cell and recording any subsequent response from the opposite cell.

The effects of GABA on the N- and P-cells were tested using a “puffer” electrode (a patch electrode with a resistance of ≈ 5 MΩ) connected to a picospritzer. The puffer electrode was positioned approximately 100 μm from the N or P soma to prevent movement artifact and a 200 msec pulse of GABA was applied at 10 psi. The membrane potential of the P-cell was maintained at −50 mV. The membrane potential of the N-cell was maintained at −60 mV in order to prevent the cell from firing when GABA was applied. The concentration of GABA within the puffer electrode was 1 M, consistent with similar studies in the leech by Sargent et al. [29]. This high concentration in the electrode was required due to the substantial amount of dilution that occurred by the time the expelled GABA reached the neuropil, which is relatively deep in the ganglion. That responses to GABA appear to arise from N- and P-cell processes in the neuropil is based on the delay between the GABA puff and the P- or N-cell response which ranged between 100–300 msec.

Post-test EPSP amplitudes and input resistance measurements were normalized relative to pre-test levels and presented as mean ± standard error. Statistical analyses using both independent and paired *t*-test or one-way analysis of variance (ANOVA) were performed to determine main effects with Newman-Keuls post-hoc tests to confirm the ANOVA results.

## Competing interests

The authors declare no competing interests.

## Authors’ contributions

AH, SY and YW carried out the electrophysiological recordings and data analysis. BDB conceived of the study and participated in its design, coordination and data analysis. BDB and AH helped to draft the manuscript. All authors read and approved the final manuscript.

## Supplementary Material

Additional file 1: Figure S1 Characterization of the lateral N-to-AP synapse. (**A**) Bath-application of the non-NMDA ionotropic glutamate receptor antagonist CNQX reduced but did not eliminate the N-to-AP EPSP. Calibration bars are 2 mV and 100 msec. (**B**) Replacement of normal leech saline with high Mg^2+^ (15 mM) saline reduced but did not eliminate the N-to-AP synapse. Calibration bars are 2 mV and 50 msec. The small, short-latency EPSP that remains following CNQX or high Mg^2+^ treatment is thought to be an electrical EPSP. (**C**) Application of high divalent saline (HiDi; 15mM Ca^2+^/18 mM Mg^2+^) reduces the decay time of the N-to-AP EPSP, consistent with removal of the later, polysynaptic components of this synaptic connection. Calibration bars are 1 mV and 50 msec. (**D**) In addition to the electrical coupling in the N-to-AP direction, there was also evidence of electrical coupling in the AP-to-N direction. Negative current injected into the AP-cell was capable of hyperpolarizing the N-cell, but positive current failed to be carried from the AP- to N-cell. The AP cell exhibits similar negative electrical coupling with the S interneuron (BDB unpublished observation). Calibration bars are 20 mV and 50 msec for the AP-cell traces (left) and 1 mV and 50 msec for the N-cell traces (right). Click here for file
