# Supplementary figures and images for: Differential modulation of nociceptive versus non-nociceptive synapses by endocannabinoids
Source: Mol Pain. 2013 Jun 1;9:26. doi: 10.1186/1744-8069-9-26 (PMC3693973; doi:10.1186/1744-8069-9-26)

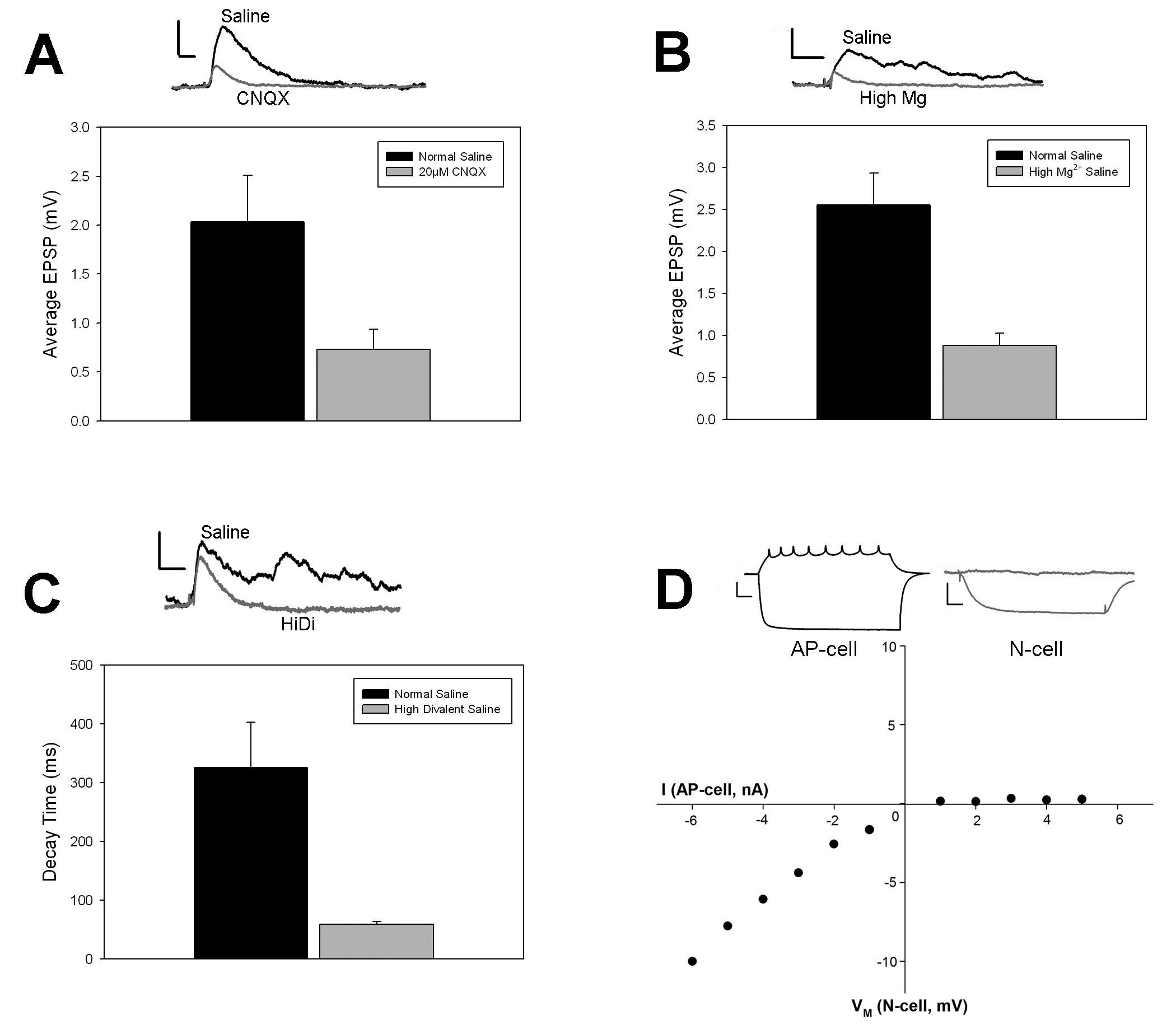

Supplement: Additional file 1: Figure S1 — Characterization of the lateral N-to-AP synapse. (A) Bath-application of the non-NMDA ionotropic glutamate receptor antagonist CNQX reduced but did not eliminate the N-to-AP EPSP. Calibration bars are 2 mV and 100 msec. (B) Replacement of normal leech saline with high Mg2+ (15 mM) saline reduced but did not eliminate the N-to-AP synapse. Calibration bars are 2 mV and 50 msec. The small, short-latency EPSP that remains following CNQX or high Mg2+ treatment is thought to be an electrical EPSP. (C) Application of high divalent saline (HiDi; 15mM Ca2+/18 mM Mg2+) reduces the decay time of the N-to-AP EPSP, consistent with removal of the later, polysynaptic components of this synaptic connection. Calibration bars are 1 mV and 50 msec. (D) In addition to the electrical coupling in the N-to-AP direction, there was also evidence of electrical coupling in the AP-to-N direction. Negative current injected into the AP-cell was capable of hyperpolarizing the N-cell, but positive current failed to be carried from the AP- to N-cell. The AP cell exhibits similar negative electrical coupling with the S interneuron (BDB unpublished observation). Calibration bars are 20 mV and 50 msec for the AP-cell traces (left) and 1 mV and 50 msec for the N-cell traces (right). [file 1744-8069-9-26-S1.jpeg]
